# Implementation of Shared Decision-Making Within Internet Hospitals in China Based on Patients’ Needs: Feasibility Study and Content Analysis

**DOI:** 10.2196/39965

**Published:** 2023-01-06

**Authors:** Tianzhi Yu, Chunjie Jin, Xiaodan Wu, Dianmin Yue

**Affiliations:** 1 Internet Hospital Tianjin Medical University General Hospital Tianjin China; 2 School of Economics and Management Hebei University of Technology Tianjin China

**Keywords:** internet hospital, shared decision-making between doctors and patients, patient needs, feasibility

## Abstract

**Background:**

Internet hospitals are developing rapidly in China, and their convenient and efficient medical services are being increasingly recognized by patients. Many hospitals have set up their own internet hospitals to provide web-based medical services. Tianjin Medical University General Hospital has established a multidisciplinary and comprehensive internet hospital to provide diversified medical services according to the needs of patients. A way to further improve web-based medical services is by examining how shared decision-making (SDM) can be carried out in internet hospital diagnosis and treatment services, thereby improving patients’ medical experience.

**Objective:**

The aim of this study was to analyze the feasibility of implementing doctor-patient SDM in internet hospital diagnosis and treatment services based on patients’ needs in China.

**Methods:**

In this study, the medical data of 10 representative departments in the internet hospital of Tianjin Medical University General Hospital from January 1 to January 31, 2022, were extracted as a whole; 25,266 cases were selected. After excluding 2056 cases with incomplete information, 23,210 cases were finally included in this study. A chi-square test was performed to analyze the characteristics and medical service needs of internet hospital patients in order to identify the strengths of SDM in internet hospitals.

**Results:**

The internet hospital patients from 10 clinical departments were significantly different in terms of gender (*χ*^2^_9_=3425.6; *P*<.001), age (*χ*^2^_36_=27,375.8; *P*<.001), mode of payment (*χ*^2^_9_=3501.1; *P*<.001), geographic distribution (*χ*^2^_9_=347.2; *P*<.001), and duration of illness (*χ*^2^_36_=2863.3; *P*<.001). Patient medical needs included drug prescriptions, examination prescriptions, medical record explanations, drug use instructions, prehospitalization preparations, further consultations with doctors (unspecified purpose), treatment plan consultations, initial diagnoses based on symptoms, and follow-up consultations after discharge. The medical needs of the patients in different clinical departments were significantly different (*χ*^2^_72_=8465.5; *P*<.001).

**Conclusions:**

Our study provides a practical and theoretical basis for the feasibility of doctor-patient SDM in internet hospitals and offers some implementation strategies. We focus on the application of SDM in web-based diagnosis and treatment in internet hospitals rather than on a disease or a disease management software. The medical service needs of different patient groups can be effectively obtained from an internet hospital, which provides the practical conditions for the promotion of doctor-patient SDM. Our findings show that the internet hospital platform expands the scope of SDM and is a new way for the large-scale application of doctor-patient SDM.

## Introduction

### Background

Shared decision-making (SDM) between doctors and patients is a decision-making process in which the doctor and patient both participate and agree on the most suitable treatment plan based on the complete information provided on the illness and relevant treatments. The doctors use their professional knowledge to elaborate on various possibilities, such as treatment options and benefits and harms, after taking into consideration the patient’s values, inclinations, and situations [1,2]. SDM between doctors and patients depends on complete information exchange and communication before the determination of the common goal of the doctors and the patients. SDM is different from the traditional biomedical model, in which the patient is only treated as a person with illness, without considering the patient’s psychological status or the social environment. SDM is also not equivalent to the biopsychosocial medical model, in which the patient’s preference is not fully considered or the medical activities are only carried out under the domination of the doctor—the diagnosis and treatment are carried out in a paternalistic manner or in an information-imparting manner. Doctor-patient SDM has been widely used in the diagnosis and treatment of different diseases, and it has proved to increase patients’ degree of participation and satisfaction in a medical service.

Internet hospitals are a new form of medical service. Through the web-based exchange of information and communication, doctors can diagnose diseases, prescribe drugs and examinations, and provide treatment guidance and health consultation for patients’ rehabilitation. Web-based medical services facilitate patients’ medical treatment, improve the efficiency of diagnosis and treatment, and reduce the cost of patients’ disease treatment [3]. At the same time, the internet hospital diagnosis and treatment information systems can record the whole process of patient diagnosis and treatment information and provide electronic medical records, treatment plans, self-diagnosis services, and other eHealth tools. Patients can receive further clarification about their disease diagnosis, treatment plan, and prognosis by using the internet hospital software.

Doctor-patient SDM is mainly implemented in clinical departments. The decision-making assistance tools corresponding to each clinical department are developed for different diseases under SDM guidance. The most suitable treatment plan for the patients is selected according to the SDM steps. For example, the implementation of a doctor-patient SDM tool in patients with vitiligo has been reported to be very successful and can be applied in other cases [4]. A doctor-patient SDM tool has also been implemented in patients with functional gastrointestinal diseases, and a variety of treatment options has been introduced and improved [5]. A study showed that carrying out doctor-patient SDM among patients with epilepsy resulted in higher-quality decision-making, more informed choices, and better treatment compliance [6]. Another study showed that the application of doctor-patient SDM among surgical patients brought about higher patient satisfaction and value recognition, which also reduced the anxiety and inner conflicts in patients [7]. The implementation of doctor-patient SDM in perinatal patients effectively reduced the dilemma of maternal decision-making [8]. The implementation of SDM for vaccine injection can effectively increase the number of vaccine injections [9].

With the development of information and communication technology, SDM between doctors and patients has gradually been implemented in the web-based medical electronic health system to serve patients with specific diseases, and decision-making aids in web versions and mobile phone versions have been developed. The operation effect shows that sufficient decision support can be obtained by using these tools. A study on a web-based decision support tool* *for patients with metastatic colon cancer showed that this tool was useful and enabled patients in effective decision-making [10]. A study on an interactive web tool for patients with dementia showed that the decision guide’s chat and personal opinion features facilitated communication between these patients and their health care workers [11]. Carolinas Asthma Coach uses SDM for child asthma [12]. The AFib app uses SDM for atrial fibrillation thromboembolism prevention [13]. e-Quit worRx is used in the treatment plan decision-making for patients in a smoking cessation program [14]. Power Up is used to record the mental status of adolescents and supports SDM during adolescent mental treatment [15]. iBdecide is a web-based tool used for adolescents on ulcerative colitis treatment, and this tool promotes patients’ active participation in doctor-patient communication [16]. A web-based SDM tool for fertility preservation in women of childbearing age with breast cancer was shown to effectively increase the ability of doctor-patient communication and patient’s independent decision-making [17]. A web-based tool that facilitated SDM between doctors and patients with regard to neoadjuvant chemotherapy use in muscle-invasive bladder cancer showed that it was beneficial to predict the treatment outcomes of these patients [18]. As an information system tool for web-based patient consultation, internet hospitals have a high degree of informatization, have large storage capacity, and are a convenient mode of communication. However, there is no research on the implementation of doctor-patient SDM in internet hospitals. This study analyzes the medical needs of internet hospital patients from the perspective of doctor-patient SDM to determine the feasibility of implementing doctor-patient SDM in internet hospitals.

Internet hospitals use network information technology to enable patients to see a doctor online. However, the patients should have visited a doctor offline first and have a basic understanding of their illness. Internet hospitals developed rapidly during the COVID-19 outbreak, and the number of internet hospitals has increased significantly since then. They have also played an important role in epidemic prevention, epidemic control, and daily web-based diagnosis and treatment [19]. This study was conducted on the internet hospital of Tianjin Medical University General Hospital. This internet hospital was self-built, while the information technology support is provided by Tianjin Careate Medical Information Co, Ltd. This internet hospital officially started operation in March 2020. This internet hospital provides services, including disease diagnosis and treatment, express drug delivery, home nursing services, discharge follow-up, and health consultation. Moreover, it specially provides medical service guidance, doctor recommendations, self-diagnosis services, disease knowledge popularization documents, complaint services, and other related services in the process of medical treatment.

**Figure 1:**
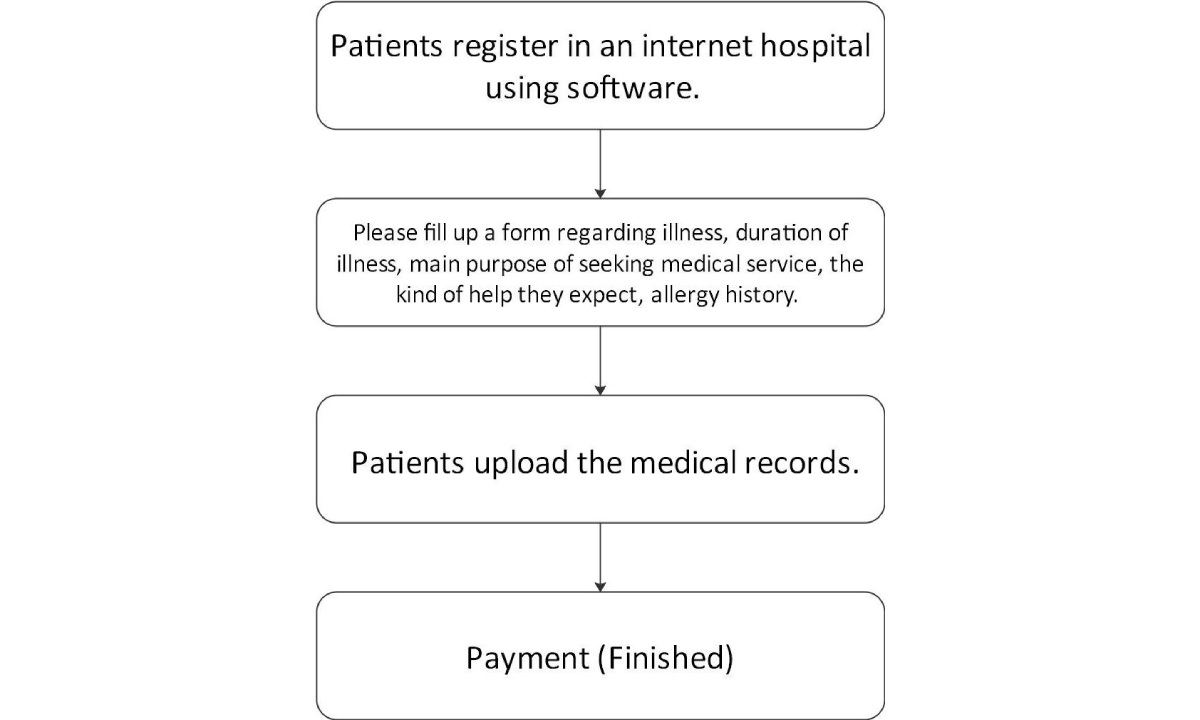
The registration flowchart of the patients in the internet hospital.

Different from other internet hospitals without brick-and-mortar hospitals, the medicines issued by the internet hospital of Tianjin Medical University General Hospital can be obtained via self-collection or express delivery. The issued examination items can be obtained offline, and the continuity of online and offline services can be realized perfectly. Figure 1 shows the registration flowchart of the patients in the internet hospital. In order to fully understand the patient’s condition and preferences, internet hospital patients are required to fill a form regarding their illnesses, duration of illness, main purpose of seeking medical service, the kind of help they expect, and allergy history and upload past medical records at the registration stage. If the patient has been visiting doctors at the hospital online or offline, the doctor can ask for the complete medical records of the patient’s outpatient, emergency, and inpatient treatments and comprehensively understand the patient’s basic situation, disease progression, and treatment plan tendencies. The use of internet hospitals by patients to seek medical treatment is based on the premise of basic early diagnosis of their own diseases, which makes SDM between doctors and patients more specific. The internet hospital also provides disease self-diagnosis services, disease popularization documents, and videos. Patients can learn about the advantages and disadvantages of different disease treatment plans through self-study. They can consult online customer service staff about which doctor they should choose for their diseases. Thus, they can choose a doctor in that specialty for the treatment of their diseases. Concurrently, patients can learn about the disease treatment plan through disease popularization videos or articles published by the doctor in advance.

The patients in internet hospitals are mainly those seeking follow-up consultation for mild and chronic diseases. Patients with acute and severe diseases are not suitable for internet hospital diagnosis and treatment. It is also relatively difficult to implement SDM between doctors and patients for patients with acute and severe diseases [20]. In view of the current popularization of medical knowledge, the improvement of education levels, the internet as the powerful search tool, the internet query function, and the medical guidance services provided by the internet hospital, the problem of asymmetric medical information between doctors and patients has gradually become balanced. Patients have a basic understanding of their own disease treatments before consultation. There is a clear direction in the selection of treatment options, which will be more conducive to the implementation of SDM between doctors and patients in the process of medical treatment in internet hospitals. Doctors master the patient’s basic situation, disease progression, and preferred treatment method before clinical consultation, thus laying the foundation for the next step to jointly decide the treatment plan with the patient.

The 3 main stages of doctor-patient SDM communication are selection talk, during which the doctor introduces the available treatment options or lets the patient know that there are more options; plan talk, during which the doctor elaborates on the advantages and disadvantages of the options available; and decision-making talk, during which the doctor discovers patient’s preferences. Afterward, they jointly choose the final treatment plan [21]. The doctors of the internet hospital basically grasp the patients’ preferences before consultation, and after consultation, they mainly conduct a plan talk and a decision-making talk according to the patient’s condition. If the internet hospital software can automatically provide decision-making aids according to the patient’s disease diagnosis during registeration, patients can know the advantages and disadvantages of various plans. The plan talk can be resolved before the consultation, and the efficiency of SDM between doctors and patients can be greatly improved.

### Objective

This paper intends to prove the feasibility of SDM between doctors and patients in internet hospitals and discusses specific implementation strategies based on the needs of the patients for medical services in an internet hospital.

## Methods

### Data Sources

The essence of SDM between doctors and patients in an internet hospital is the perfect match between doctors’ professional knowledge and patients’ preferences for disease treatment needs. The most appropriate method to solve the actual medical needs is selected through the steps of understanding, communication, and decision-making. The Ottawa doctor-patient SDM framework is divided into the following three parts: decision-making needs, decision-making results, and decision-making support. Of these, decision-making needs are the primary issue to make clear [22]. In this paper, the medical data of 10 representative departments in the internet hospital of Tianjin Medical University General Hospital from January 1 to January 31, 2022, were extracted as a whole. The selection of 10 departments took into account the characteristics of diseases and differences in treatment methods, service objects, and service mode between internal medicine departments and surgery departments. Accordingly, 5 departments focusing on internal medical treatment and 5 departments focusing on surgical treatment were selected. The age and gender of the patients were considered. The selected departments were pediatrics, obstetrics and gynecology, cardiology, neurology, nephrology, endocrinology, orthopedic surgery, neurosurgery, general surgery, and lung tumor surgery, with a total of 25,266 cases. After excluding 2056 cases with incomplete information, 23,210 cases were finally included in this study. According to the needs reported by the patients when they registered in the internet hospital, the communication records of doctors and patients, and the analysis of the final medical records, the patients in the internet hospital were divided into 6 situations, as follows.

First, patients are very aware of their disease conditions and treatments. They have clear purposes for medical treatment in the internet hospital, such as patients with diabetes, hypertension, hypercholesterolemia, hyperuricemia, cerebral infarction, or Alzheimer disease, among others.

Second, patients know the illness but do not understand the progress of the disease or the importance of regular re-examination. Patients need re-examination because of new symptoms but do not know the specific re-examination items, such as re-examination after cardiac stent surgery, re-examination after lung cancer surgery, regular re-examination for patients with hyperthyroidism, and regular examination of pregnant women.

Third, patients have doubts about the treatment effect after the diagnosis and request to further confirm the direction of diagnosis and treatment, such as requiring adjustment for the type or dosage of medication and seeking other treatment options. Patients’ diseases include stunted growth, chronic kidney disease, and hyperprolactinemia, among others. The short-term disease treatment effect is not obvious. As such, the patients require more effective treatment.

Fourth, the patient has undergone a preliminary diagnosis by a doctor. After that, the relevant test results have come out. They need the doctor to further explain and formulate the next diagnosis and treatment plan. For example, they need the doctor to explain the computed tomography report, magnetic resonance image report, blood report, urine report, feces report, pathology report, physical examination report, and other functional examination reports.

Fifth, the patients do not understand the health condition, disease symptoms, or other symptoms that are suspected to be caused by the existing disease. In addition, it is not strictly possible to determine if the patients who seek medical treatment in the internet hospital are return-visit patients. Many patients have uncomfortable symptoms, and they choose web-based consultation to know the reasons for their symptoms as well as treatment plans. The symptoms include dizziness, insomnia, tinnitus, cough, chest tightness, itching, and pain.

Sixth, the patient’s disease has been diagnosed, and the patient needs to be hospitalized. The prehospitalization preparation work should be done through the internet hospital, or the examinations required for hospitalization should be subscribed in advance. The examination results will be reported to the doctor directly before hospitalization in order to reduce the actual hospitalization length of stay.

According to patients’ understanding of their own diseases, the service needs of internet hospital patients were divided into drug prescription, examination prescription, medical record explanation, medication guidance, prehospitalization preparation, disease follow-up consultation (unspecified purpose), treatment plan consultation, initial diagnosis of symptoms, and follow-up consultation after discharge. A chi-square test was performed to analyze the patient characteristics and the distribution of medical needs. This test analyzes whether there were differences in the related data indicators of the internet hospital patients among different departments.

### Ethical Considerations

This study was reviewed by the ethics committee of the Tianjin Medical University General Hospital (approval number IRB2022-WZ-210). This research on patient services in an internet hospital was an analysis of patients’ medical behavior data, which did not involve human experiments or compensation. The Tianjin Medical University General Hospital approved the study data collection from the hospital information system. In order to protect the privacy of patients, some data were deleted during extraction, such as the patient’s name, address, and contact information, as well as other information that could be used to identify the patient. To the researchers, the patients were anonymous or unidentified. The patient-related data sets met the ethical requirements. The research team complied with informed consent guidelines and have adhered to local, national, regional, and international law and regulations regarding the protection of personal information, privacy, and human rights. The ethics committee of the Tianjin Medical University General Hospital approved this study after review.

## Results

Table 1 and Table 2 show the characteristics of the patients and their service needs in the internet hospital, respectively. The data show that the internet hospital can supply the diagnosis and treatment services in different clinical departments to meet the needs of different patient groups.

**Table 1:** Characteristics of the patients using the services of an internet hospital (N=23,210).

Characteristics		Pediatrics, n	Endocrinology, n	Nephrology, n	Cardiovascular, n	Neurology, n	Lung tumor surgery, n	Obstetrics and gynecology, n	Orthopedic surgery, n	General surgery, n	Neurosurgery, n
**Gender**											
	Male	1137	132	989	2097	1421	360	N/A^a^	377	98	364
	Female	1015	3586	1025	1987	1731	470	3969	597	193	473
**Age (years)**											
	≤15	2152	22	2	8	9	N/A	6	10	4	4
	>15 to ≤30	N/A	1150	321	242	311	30	1332	189	71	123
	>30 to ≤45	N/A	2118	725	1138	651	161	2126	347	122	238
	>45 to ≤60	N/A	963	508	1161	732	294	381	237	46	238
	>60	N/A	654	458	1535	1449	345	124	191	48	234
**Payment**											
	Medical insurance	90	3417	1342	3119	1937	446	2233	564	181	491
	Self-paid	2062	1490	672	965	1215	384	1736	410	110	346
**Region**											
	Local patient	1802	3991	1652	3480	2310	570	2939	719	234	632
	Nonlocal patient	350	916	362	604	842	260	1030	255	57	205
**Duration of illness (days)**											
	≤7	1421	1340	694	1303	857	260	2314	357	149	332
	>7 to ≤30	328	410	157	441	289	117	489	156	48	105
	>30 to ≤183	162	764	323	573	558	178	516	230	43	154
	>183 to ≤365	82	514	220	360	263	97	210	62	16	52
	>365	159	1879	620	1407	1185	178	440	169	35	194

^a^N/A: Not applicable.

**Table 2:** Patients’ medical service needs in the 10 clinical departments (N=27,445).

Service needs	Pediatrics, n	Endocrinology, n	Nephrology, n	Cardiovascular, n	Neurology, n	Lung tumor surgery, n	Obstetrics and gynecology, n	Orthopedic surgery, n	General surgery, n	Neurosurgery, n
Prescribing drugs	147	743	543	1421	1099	44	243	116	26	166
Prescribing examinations	113	526	221	262	119	78	319	41	30	52
Explanation of medical records	243	1643	746	696	387	373	1229	188	43	158
Symptoms at initial diagnosis	1131	293	97	457	843	45	795	386	123	177
Medication guidance	369	1051	186	856	498	33	414	48	28	498
Prehospitalization preparation	11	21	90	68	35	40	94	30	8	25
Treatment plan consultation	242	485	171	414	346	166	578	248	56	120
Follow-up consultation after discharge	76	186	82	458	165	234	240	64	15	224
Disease follow-up consultation (unspecified purpose)	177	716	158	220	126	40	616	26	4	27

Table 1 shows that there were significant differences in the gender (*χ*^2^_9_=3425.6; *P*<.001), age (*χ*^2^_36_=27,375.8; *P*<.001), mode of payment (*χ*^2^_9_=3501.1; *P*<.001), geographic distribution (*χ*^2^_9_=347.2; *P*<.001), and duration of illness (*χ*^2^_36_=2863.3; *P*<.001) among the patients using the services of the internet hospital. The majority of the patients in these internet hospitals were female, accounting for 64.77% (15,035/23,210) of the sample population. The proportion of patients aged 30 to 45 years was the highest, accounting for 32.85% (7624/23,210) of the sample population. Patients younger than 60 years accounted for 78.29% (18,172/23,210), and older adults accounted for 21.71% (5029/23,210) of the sample population. Patients covered by medical insurance accounted for 59.54% (13,820/23,210), and patients who paid their own expenses accounted for 40.46% (9390/23,210) of the sample population. Patients in Tianjin accounted for 78.97% (18,329/23,210), and patients from other places accounted for 21.03% (4881/23,210). Patients with illness for less than 7 days accounted for 38.89% (9027/23,210), and patients with illness for more than 7 days accounted for 61.11% (14,183/23,210) of the sample population. Patients whose duration of illness was longer than 1 year accounted for 26.99% (6266/23,210) of the sample population. Internet hospitals provide a convenient way for web-based medical treatment and medical insurance settlement. Female and young patients were more likely to use web-based health care. The local patients were also willing to choose this convenient method of medical treatment. In particular, patients with chronic diseases need multiple consultations; therefore, internet hospitals are the best choice for consultation for such patients. With the development of internet hospitals, patient reception and the service radius will become larger. Internet hospitals have a large number of patients, and they can especially facilitate the long-term diagnosis and treatment of patients with chronic diseases, which is conducive to the large-scale implementation of SDM.

Table 2 shows that there is a significant difference in the internet hospital service needs of patients in different departments (*χ*^2^_72_=8465.5, *P<.*001). Among the service needs of patients, report explanation, drug prescription, preliminary symptom diagnosis, and medication guidance ranked as the top 4 needs, which accounted for 67.51% (18,528/27,445) of the total demand. Patients who choose internet hospitals for treatment have a basic understanding of their disease. They directly choose drug prescriptions and examination prescriptions, make appointments for hospitalization, seek medication guidance, and seek follow-up consultation after discharge. They account for 45.39% (12,456/27,445) of the total demand. Patients who do not understand their own discomfort symptoms are basically patients with mild symptoms. They also seek medical treatment through the initial diagnosis of symptoms, and these patients account for 15.84% (4347/27,445) of the total demand. In addition, patients who do not know their own conditions and only rely on doctors to issue treatment plans unilaterally account for 17.99% (4936/27,445) of the total demand. Due to the continuity of disease diagnosis and treatment, patients may not complete the whole diagnosis and treatment process during only 1 consultation. Internet hospitals provide subsequent diagnoses and treatment platforms. Internet hospitals can easily prescribe drugs, interpret reports, provide drug guidance, diagnose symptoms, and follow up on consultation after treatment. The analysis of patients’ service needs showed that internet hospitals can provide diversified diagnoses and treatment services, laying the practical foundation for the feasibility study of doctor-patient SDM in internet hospitals.

## Discussion

### Principal Findings

Internet hospitals provide multidisciplinary medical services and achieve full coverage of different medical service needs. Even local patients are willing to use web-based diagnosis and treatment services. Internet hospitals provide a convenient way to seek medical service. Web-based medical treatment reduces transportation costs and waiting time costs. Online and offline medical service information links make services more efficient and timely. Moreover, internet hospitals provide online medical insurance reimbursement payment, which more effectively solves the problem of patient payment. China’s internet hospitals are a new medical mode that has developed rapidly in the past 5 years. Previous studies, such as those regarding Carolinas Asthma Coach [12], the AFib app [13], e-Quit worRx [14], Power Up [15], and iBdecide [16], focused on the application of doctor-patient SDM through a disease or a disease management software. To date, no doctor-patient SDM app has been used in an internet hospital. Internet hospitals provide a good platform and a big patient group for promoting doctor-patient SDM apps. The internet hospital is also a new way for the large-scale application of doctor-patient SDM. The data on patients’ service needs provided by internet hospitals show that it is favorable to implement doctor-patient SDM in internet hospitals. However, it is necessary to consider the characteristics of the patients and service needs of the internet hospital, make full use of the advantages of internet hospitals (eg, online communication, storage and query functions, and convenient medical services for patients), and carry out the targeted promotion and application of doctor-patient SDM.

There are many advantages of doctor-patient SDM in internet hospitals. Patients in internet hospitals are mainly patients with chronic diseases and mild diseases. Especially for patients whose service needs are drug prescriptions, examination appointments, prehospitalization preparations, medication guidance, and follow-up consultations after discharge, decision-making is relatively easy and simple, since the purposes are clear. For medical needs, such as medical record explanation, disease follow-up consultation (unspecified purpose), treatment plan consultation, and initial symptom diagnosis, the decision-making process is relatively complicated due to the existence of unknown conditions and the unclear purpose of the patient’s consultation. However, the support of the eHealth information in the internet hospital information system has a great advantage over offline doctor-patient SDM. First of all, most of the patients in internet hospitals are younger than 60 years, and parents of children patients should communicate with the doctors online to lay a foundation for effective communication. Second, the patient’s condition and patient’s preferences can be grasped in advance. Compared with the on-site decision-making process of offline outpatients or inpatients, patients in internet hospitals can understand the plan and explain their preferences clearly. Doctors can understand the basic conditions of their patients before they receive the patients, which greatly improves the efficiency of doctor-patient communication. Lastly, the illnesses of patients who make decisions are relatively simple. Compared to patients with acute and critical diseases, patients in internet hospitals are generally those with mild, long-term, or chronic diseases; therefore, the time for communication is sufficient.

The implementation of SDM should result in a grasp of the patients’ needs. According to the analysis of patient service needs in internet hospitals, the needs of patients in different departments are significantly different. Thus, differentiated strategies should be adopted according to the needs of patients in different departments in order to promote SDM between doctors and patients. From the perspective of the needs of patients in an internet hospital, the patients who visit a doctor are divided into 2 groups. The first group comprises patients with chronic diseases and have made multiple consultations. They have clear preferences for treatment plans and their purpose. They already know what medicine and examination items should be prescribed. In this case, it is easy for doctors to judge patients’ preferences and choose a plan based on past diagnosis and treatment records, as the doctor’s and patient’s treatment plans are basically the same. For example, doctors can directly prescribe drugs or examinations according to patients’ requirements or conduct prehospitalization preparations and follow-up consultations after discharge. The second group comprises patients whose diagnosis, treatment, and re-examination are not clear. The patient cannot make a direct demand for medical treatment. The doctor needs to make a judgment based on the medical records provided by the patient and further confirm the disease diagnosis. The medical service needs of these patients include report explanation, medication guidance, treatment plan consultation, initial symptom diagnosis, and disease follow-up consultation (unspecified purpose). The focus of doctor-patient SDM in internet hospitals should be on the second group of patients.

SDM tools should be developed for doctors and patients of internet hospitals. There is no clear definition for doctor-patient SDM aid tools. They can be understood as tools that integrate articles, pictures, and videos, among other media, that are related to disease diagnosis, treatment, and rehabilitation. The main content expressed should be the adaptation of different treatment plans to the intended population. According to the classification of common diseases, clinical departments should formulate differential disease diagnosis plans, examination and testing plans, treatment plans, medication plans, regular re-examination plans, and rehabilitation plans. Clinical departments can send medical service information or supply self-service inquiry functions to patients via internet hospital software. Clinical departments can also focus on formulating relevant plans for patients’ reference and decision-making according to the differentiation of patients’ needs. With the help of the information system, doctors in internet hospitals can not only conveniently query the historical medical records of patients but also easily provide corresponding auxiliary tools for SDM to patients. Patients can also easily query the medical records of previous visits, the voice or written records of SDM communication between doctors and patients, the videos of doctors explaining the diseases they are specialized in, and popular science articles. Thus, the awareness of disease conditions and treatment options is enhanced. It is more helpful if patients take the initiative to participate in SDM between doctors and patients. Given that the service needs of internet hospital patients are mainly the explanation of reports, the prescription of drugs, the initial diagnosis of symptoms, and guidance on medications, the development of auxiliary tools should focus on the differential diagnosis of diseases, treatment plans, and medication guidance.

Many factors influence SDM between doctors and patients in an internet hospital. Hospitals, doctors, and patients are the main driving forces for the implementation of doctor-patient SDM in internet hospitals. Hospitals should incorporate doctor-patient SDM into internet hospital diagnosis and treatment and promote the implementation of doctor-patient SDM at the policy level. Doctors are the determining factor in the SDM between doctors and patients. They should formulate a doctor-patient SDM tool based on the type of disease and apply it to disease diagnosis and treatment activities. Under the guidance of doctors, patients should make full use of doctor-patient SDM tools and combine their own preferences with SDM to determine the most suitable diagnosis and treatment plan. Hospitals, doctors, and patients should form a good cooperation mechanism to effectively promote the SDM between doctors and patients. Internet hospitals provide a platform for patients and doctors to communicate. Carrying out SDM between doctors and patients can improve the quality of medical services and the satisfaction of patients, which can indirectly improve patient loyalty. SDM also results in the formation of a continuous, stable, and harmonious doctor-patient service relationship, as doctors can easily monitor the disease progression of patients; in fact, SDM is more conducive to disease treatment.

### Limitations

This study has some limitations. First, we only selected internet hospital data from representative clinical departments and analyzed the related needs. We did not cover all disciplines, and there is a certain one-sidedness in the needs of patients in internet hospitals. Second, the development of aid tools and the information platforms suitable for internet hospital SDM need to be further studied, especially in terms of how to embed the auxiliary tools for doctor-patient SDM in the internet hospital information platform. Both artificial intelligence and the robot dialogue method can be used to solve the issues with the first stage (selection talk) and second stage (plan talk) of doctor-patient SDM, which will be the contents of future research.

### Conclusions

This study proves the feasibility of implementing SDM in internet hospitals and provides some implementation strategies. Internet hospitals should promote SDM between doctors and patients at the overall level. The clinical departments should jointly formulate standardized auxiliary tools for SDM between doctors and patients for common diseases, provide these tools to patients for learning and consideration in advance, and guide patients to carry out SDM to improve decision-making efficiency. Different from previous studies, this study focuses on the application of SDM in web-based diagnosis and treatment rather than on a disease or a disease management software. Through the function of this web-based platform, the application scope of SDM can be expanded, and a new field of SDM application can be developed. Thus, internet hospitals provide a new way for the large-scale application of doctor-patient SDM.

## Abbreviations

SDM: shared decision-making

## Data Availability

The data analyzed during this study are included in this published paper. The raw data sets are not publicly available because of patient privacy protection.
